# Individual differences in learning correlate with modulation of brain activity induced by transcranial direct current stimulation

**DOI:** 10.1371/journal.pone.0197192

**Published:** 2018-05-21

**Authors:** Brian Falcone, Atsushi Wada, Raja Parasuraman, Daniel E. Callan

**Affiliations:** 1 Center of Excellence in Neuroergonomics, Technology, and Cognition (CENTEC), George Mason University, Fairfax, Virginia, United States of America; 2 Center for Information and Neural Networks (CiNet), National Institute of Information and Communications Technology (NICT), Osaka University, Osaka, Japan; Shanghai Mental Health Center, CHINA

## Abstract

Transcranial direct current stimulation (tDCS) has been shown to enhance cognitive performance on a variety of tasks. It is hypothesized that tDCS enhances performance by affecting task related cortical excitability changes in networks underlying or connected to the site of stimulation facilitating long term potentiation. However, many recent studies have called into question the reliability and efficacy of tDCS to induce modulatory changes in brain activity. In this study, our goal is to investigate the individual differences in tDCS induced modulatory effects on brain activity related to the degree of enhancement in performance, providing insight into this lack of reliability. In accomplishing this goal, we used functional magnetic resonance imaging (fMRI) concurrently with tDCS stimulation (1 mA, 30 minutes duration) using a visual search task simulating real world conditions. The experiment consisted of three fMRI sessions: pre-training (no performance feedback), training (performance feedback which included response accuracy and target location and either real tDCS or sham stimulation given), and post-training (no performance feedback). The right posterior parietal cortex was selected as the site of anodal tDCS based on its known role in visual search and spatial attention processing. Our results identified a region in the right precentral gyrus, known to be involved with visual spatial attention and orienting, that showed tDCS induced task related changes in cortical excitability that were associated with individual differences in improved performance. This same region showed greater activity during the training session for target feedback of incorrect (target-error feedback) over correct trials for the tDCS stim over sham group indicating greater attention to target features during training feedback when trials were incorrect. These results give important insight into the nature of neural excitability induced by tDCS as it relates to variability in individual differences in improved performance shedding some light the apparent lack of reliability found in tDCS research.

## Introduction

The passing of low intensity electric current through the scalp to underlying brain tissue by transcranial direct current stimulation (tDCS) has been used extensively in both research and clinical settings to improve performance and learning on various perceptual, motor, and cognitive tasks as well as for treatment of various mental and neurological disorders [1–5]. TDCS is thought to modulate neuronal excitability and improve learning by long-term potentiation/depression LTP/LTD [2, 6–13]. Anodal stimulation is thought to promote cortical excitability whereas cathodal stimulation is thought to suppress cortical excitability [11, 14]. Despite the considerable number of studies showing modulatory effects of tDCS on human behavior in healthy individuals there is still dispute of its effects on the brain as well as its efficacy in terms of its reliability [15–22].

To better understand the underlying effects that tDCS has on the brain several studies have been conducted using tDCS in conjunction with non-invasive brain recording such as electroencephalography EEG [23–28], magnetoencephalography MEG [29–31], functional near infrared spectroscopy fNIRS [32], and functional magnetic resonance imaging fMRI [33–50]. These studies have shown considerable variability in the effects of tDCS on brain activity and connectivity dependent on presence or absence of a task, stimulation site, polarity of stimulation, amplitude of stimulation, timing of stimulation, and nature of the task under investigation. The general findings of this research include the following: tDCS influences brain networks with regions both proximal and distal to the stimulating electrodes including those in subcortical regions [37–39, 48–51]. The extent of modulation of brain activity and connectivity is task dependent [37, 52]. It is interesting to point out that while some studies [44] find an enhancement in brain activity as a result of anodal stimulation that is related to facilitation of behavioral performance (consistent with the idea of tDCS induced excitability leading to LTP), there are others [40, 41, 45] that find a reduction in brain activity consistent with the idea of enhanced efficiency.

In an attempt to better understand whether apparent discrepancies in the effectiveness of tDCS is related to individual differences in its induced neural modulation, we investigated both the after-effects of tDCS as well as the concurrent effects during task training using simultaneous fMRI-tDCS. For this study, we employed a visual search task that simulates real world conditions. Participants were to identify the presence or absence of a specific red truck amongst distractors in an urban environment through a simulated unmanned aerial vehicle (UAV) video-feed. We selected the right posterior parietal cortex rPPC as our site of anodal stimulation based on previous behavioral tDCS studies that have shown that stimulation of this region results in performance changes on a variety of spatial attention tasks related to visual search [53–55].

Visual search is intimately related to both bottom-up (saliency driven) and top-down (goal-directed) processes as they interact to reorient attention to behaviorally relevant stimuli [56, 57]. The rPPC is crucially involved in visual search [57], as it is included in both the dorsal fronto-parietal and the right ventrofrontal temporoparietal networks. In a study conducted by [38] Ellison et al. (2014), it was shown that tDCS could be used to reveal the relationship between the critical nodes within these networks during visual search. Cathodal stimulation was applied to the right PPC for 15 minutes before participants performed a visual search task during fMRI scanning. Results showed that cortical inhibition of the rPPC induced by cathodal stimulation resulted in a decrease in activation in frontal brain regions, specifically, the frontal eye fields (FEF) in the premotor cortex. These results make it clear that stimulation of the rPPC can result in activation changes in brain areas throughout the entire attention network involved in visual search in addition to the area being stimulated. This suggests that we can expect to see a similar pattern in our own results.

It has been previously posited that on complex visual perceptual detection tasks, such as visual search, tDCS enhances learning mechanisms through an improvement in attention [58], which is facilitated most likely by LTP induced synaptic strengthening of the attentional network. Resting state networks in brain regions involved with focused attention have been found to show greater connectivity after tDCS [47]. This suggests that increased attention results in more efficient processing of target features and a suppression of distractor features which in turn leads to enhanced encoding during learning [2, 58, 59]. The dopaminergic system including the substantia nigra and basal ganglia is thought to facilitate LTP [60] and may be an important component of attentional learning.

Towards the comprehensive understanding of the specific induced modulation that tDCS has on brain activity during learning and performance of a complex visual search task, we devised an integrative experimental design that enables us to investigate: individual differences in neural after-effects of tDCS as related to variable facilitation in behavioral performance, and the specific concurrent neural effects of tDCS on brain processes related to complex perceptual learning. We used error feedback training, in which visual and auditory feedback indicating the true target is presented following each response. This scheme facilitates subjects to acquire relevant target features and allows us to focus on brain activity during the time period where the greatest amount of learning is expected to take place. The primary goals of the experiment are the following: 1. To determine individual differences in behaviorally related changes in brain activity post- relative to pre-training that is specific to the tDCS stim over the sham group. This is accomplished by correlating differential brain activity from post- relative to pre- training with each individual’s improvement in visual search performance after training and comparing this correlation between real and sham tDCS. Consistent with the hypothesis of tDCS-induced excitability leading to LTP, it is predicted that specific effects of tDCS will be seen in the form of a positive relationship between improvement in behavioral performance and increased brain activity in the rPPC (site of stimulation) and/or in related brain regions involved with visual search. In other words, individuals in the tDCS stim group who display larger increases in brain activity will also have larger improvements in visual search performance following training compared to the tDCS sham group. These results would suggest that tDCS stimulation is enhancing learning selectively for the tDCS stim group. Conversely, we may also see a reduction in activity in brain regions involved with visual search with improvement in behavioral performance for the tDCS stim group over the tDCS sham group reflecting more efficient processing (consistent with [40–41, 45]). 2. To determine the effect that tDCS has on cortical activity that may enhance the likelihood of learning. In accomplishing this goal, we examine the difference in brain activity during target feedback of incorrect versus correct trials between real tDCS stim and tDCS sham groups while undergoing simultaneous fMRI scanning. It is hypothesized that the tDCS stim group will show specific differences in activity related to attention and learning of the visual search task via target feedback. We focus on target-error feedback (feedback following an incorrect response in which a visual cue is given as to the location of the target on the scene allowing for relevant features to be evaluated) because of its involvement in attention, learning and potential link in enhancing behavioral performance. 3. To determine the overlap in tDCS specific brain regions for these two analyses (Conjunction of results from 1 and 2 above). It is hypothesized that this overlap may reflect brain regions involved with tDCS-induced training effects that are related to after-effects correlated with individual differences in performance improvement. Fulfilling the goals of this experiment will help elucidate the neural processes underlying behaviorally related modulation in performance induced by tDCS to better understand individual differences in its efficacy likely responsible for the low degree of reliability amongst tDCS studies in healthy adults.

## Methods

### Participants

The participants consisted of 28 Japanese adults (14 males, 14 females) aged 18–25 years (mean = 20.7) from Osaka University. Participants were screened and excluded for having a history of head injuries or concussions, current or previous history of mental, neurological, alcohol or drug abuse disorders, or current medication affecting central nervous system function. Subjects without normal vision were given MRI compatible glasses to correct their vision to normal before the study was conducted and all subjects were right-handed. Participants were randomly assigned to the real tDCS (which will be referred to as the “stim group” from this point forward) or sham group. There were 14 individuals (7 females and 7 males) in the tDCS stim group (mean age = 22.1 years) and 14 individuals (7 females and 7 males) in the tDCS sham group (mean age = 21.3 years). Several subjects were not included in this study as a result of pressure pain caused by the tight fit of the headphone within the head coil or below chance performance on the post-training session. In the tDCS stim group there were originally 18 subjects and 4 were removed from the study. For the sham group, there were originally 17 subjects and 3 were removed from the study. Subjects gave written informed consent. The experimental procedures were approved by the National Institute of Information and Communications Technology NICT Human Subject Review Committee and were carried out in accordance with the principles expressed in the WMA Declaration of Helsinki. The person giving the instructions was blind with regard to the group membership of the participant. Therefore, this is a double-blind study.

### Unmanned aerial vehicle (UAV) visual search task

**Fig 1:**
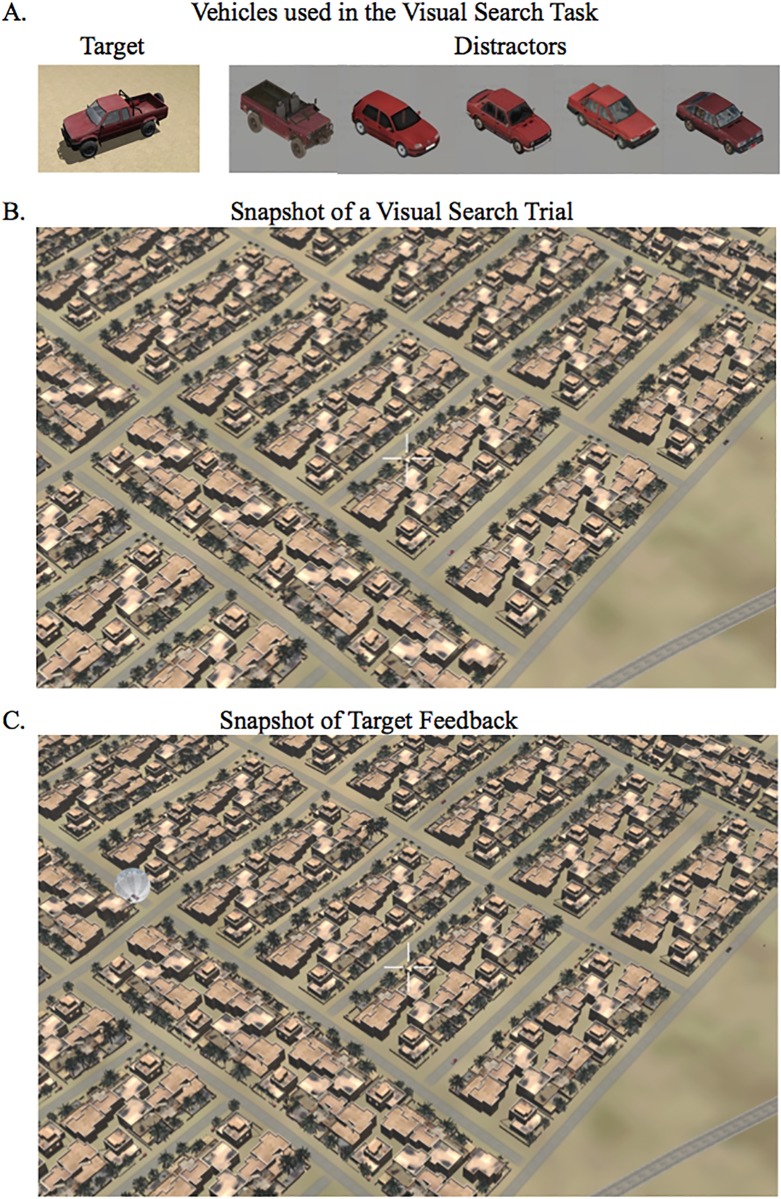
Example of UAV visual search task. A) Image of red truck target and five possible distractors. All distractors were set to share a similar feature with the target (red color) in order to increase difficulty and could be present multiple times or not at all within each trial. A total of five vehicles were present per trial, including the target for target-present trials. B) Snapshot of a visual search trial used for both test and training sessions. C) Snapshot of target feedback from the training session. Following participant response, the sphere would appear over the area where the target was present in target-present trials.

A high-fidelity battlefield simulator and training platform, Virtual Battlespace 2 (VBS2), was used to program the UAV visual search task. Participants viewed a 3D virtual Middle Eastern urban environment on a monitor from the perspective of a video feed from an MQ-9 Predator unmanned aerial vehicle. In each trial, the UAV camera was locked to the central point of the area to be surveyed as the UAV loitered in a circular path around this point. The goal of the task was based on a search and rescue mission that required participants to locate a red pickup truck located in the search area amongst buildings and other similar looking distractor vehicles. In each trial, there were 5 non-moving vehicles distributed throughout the search area, one of which could be the red truck (Fig 1A). These vehicles were located in a variety of areas such as on roads, in parking lots, next to walls or buildings or out in the open, away from any man-made structures (Fig 1B). The task was designed so that as the UAV loitered in a circle around the search area, all vehicles would remain in constant view despite a continually changing view angle. The visual search portion of each trial lasted 10 seconds where the participants searched the area looking for the target and were required to make a button press indicating whether the search area contained a target or not. If they did not respond by the end of the trial, these trials were removed from the analysis. The justification for removing these trials was that we were mainly interested in focusing on the time in which the visual search decision was made. We believe that the button response is a good indicator of this time. There was approximately a 2 second inter-stimulus interval (ISI) before the start of each visual search trial, which consisted of a black screen and white crosshair fixation.

All distractor vehicles in this task were chosen to share one particular feature with the target vehicle, which is the color red (see Fig 1A for pictures of the target and distractor vehicles). This allowed us to easily manipulate the difficulty of the task based on the flying altitude of the UAV because at higher altitudes it becomes exceedingly difficult to distinguish between vehicles of the same color. Through initial pilot testing, the permanent flying altitude of the UAV for the experiment was chosen based on the altitude that yielded an average of ~66% performance accuracy with no prior visual search training and also when sitting at a distance of 1.2 meters from the display. This was to ensure that the task was sufficiently difficult with plenty of room for improvement but also not so difficult that it resulted in chance level performance where early improvements would be due to initial task experience rather than tDCS-elicited cognitive enhancement. The distance of 1.2 meters was used in the pilot study as this was also the distance of the display from the participant in the MRI scanner.

**Fig 2:**
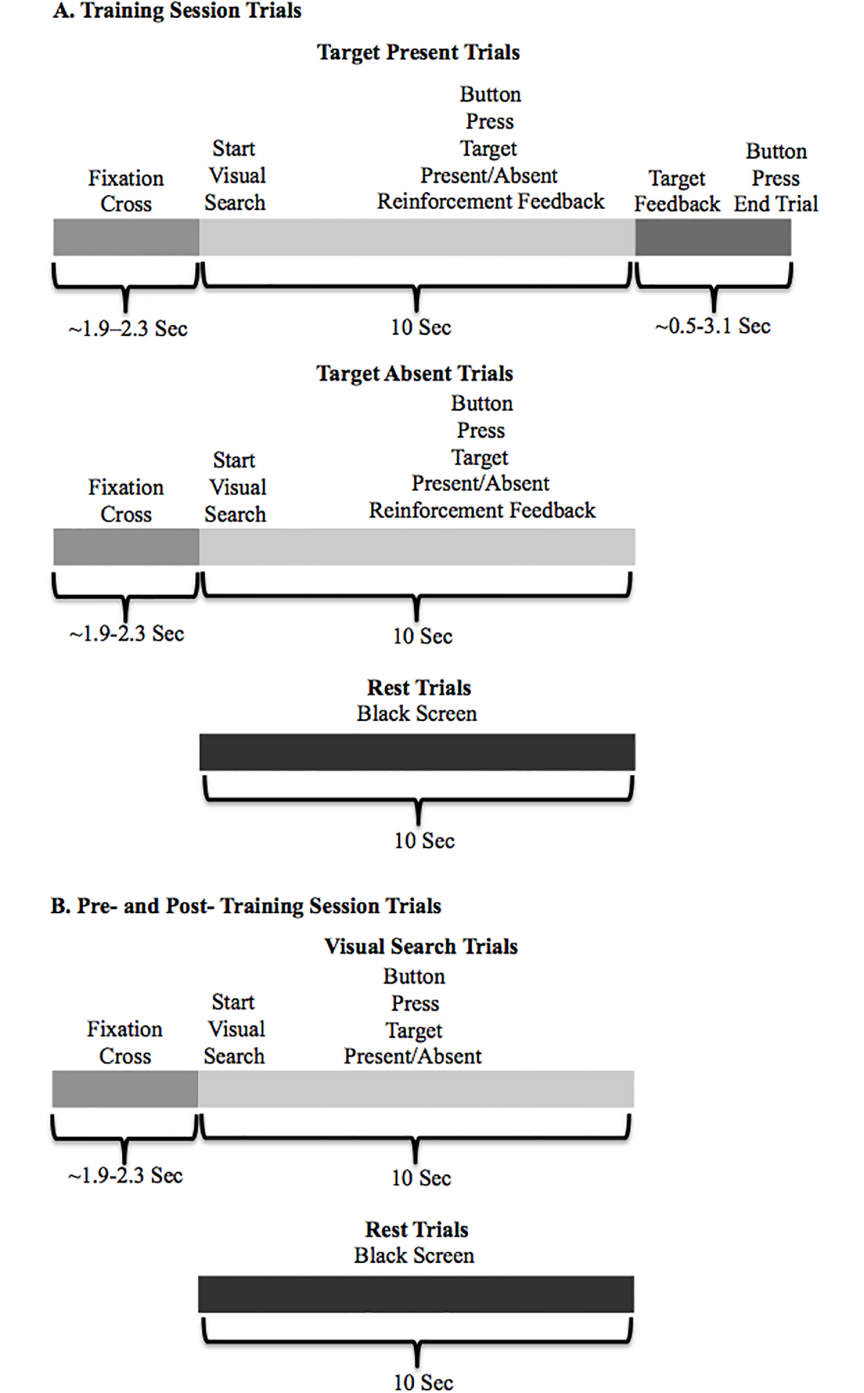
Example of UAV visual search task. Timing of the different types of trials in the A. Training session (simultaneous tDCS stim or sham) and B. Pre- and post-training sessions.

There were three sessions: a pre-training session that did not provide performance feedback, a training session, which provided immediate feedback after each response as well as target feedback after the trial, and a post-training session with no feedback. Each session consisted of 60 visual search trials and 15 baseline control trials in which subjects looked at a black screen. See Fig 2 for the timing aspects of each of the trial types for A. the training session and B. the pre- and post-training sessions. The visual search portion of the trial was 10 seconds long. Half of the visual search trials contained a target (red truck) (see Fig 1B for a snapshot picture of a visual search trial). In the feedback trials, a sound would play immediately after the participant responded by button press, which would indicate whether or not they answered correctly. They would hear a ‘ding’ sound-effect for correct responses and a ‘buzz’ for incorrect responses. For target present trials only, a transparent white sphere would appear over the target at the end of the 10-second trial (See Fig 1C for a snapshot picture of an example of the target feedback). This sphere was used to draw attention to the actual location of the target, which allowed effective target feedback and also gave the participant the opportunity to study the features of the target and to use this knowledge for future trials. The sphere would remain over the target and the next trial would not begin until the participant responded with a button-press a second time indicating whether or not they saw this target. If they indicated that the target was present with their first response but had actually been looking at a distractor when the sphere appeared highlighting the real target they would respond in the negative with the second button-press. This allowed us to determine true “hits” from guessing. If they had missed the target and indicated that the target was absent with their first response they would simply respond in the negative for the second button-press.

### Behavioral data analysis

Performance measures and response times were collected for each of the three sessions. Performance measures included: percent correct, hit rate, and false alarm rate. Response time measures included: 1. Response time for all trials (RT all search); 2. Response time for hit and correct rejection trials (RT HT&CR Search); 3. Response time for target present trials (RT TP trials Search); 4. Response time to target for target present trials (RT TP trials Target). The “RT TP trials Search” and “RT TP trials Target” measures were only analyzed for the training session to correspond with an fMRI analysis looking at the effects of feedback (see FMRI Data Collection and Analysis below) as visual target feedback only occurred in the training session and on target present trials. All measures (excluding RT TP trials Search & RT TP trials Target) were analyzed using between subjects t-tests (stim vs. sham) to identify any significant differences. This was done for each session as well as for gain scores from the pre-training session to the post-training session.

### FMRI data collection and analysis

All 3 sessions were conducted within a Siemens 3T Trio Scanner using a 32-channel head coil at the Center for Information and Neural Networks NICT Osaka University. The functional T2* weighted images were acquired using a gradient echo-planar imaging EPI sequence with a repetition time of 2 seconds (TR = 2). Each scan consisted of 30 interleaved axial slices (3x3x4mm) covering the brain and cerebellum. For sessions 1 and 3, 454 scans (~15 minutes) were taken for each session. For session 2 approximately 475 scans (~16 minutes) were taken that varied depending on the participant’s response time on the feedback trials. An additional 150 scans (5 minutes) measuring resting-state activity were collected after each experimental session. The analysis and results of the resting state activity are presented in a separate study [37]. Images were preprocessed using programs within SPM8 (Wellcome Department of Cognitive Neurology, UCL). Images were realigned, unwarped, spatially normalized to a standard space using a template EPI image (2x2x2 mm voxels), and were smoothed using an 8x8x8 mm FWHM Gaussian kernel. Regional brain activity for the various conditions was assessed using a general linear model employing an event-related design in which a modeled canonical hemodynamic response function (HRF) was convolved with the event onset predictors. Auto-regression was used to correct for serial correlations. High pass filtering (cutoff period 128 s) was carried out to reduce the effects of extraneous variables (scanner drift, low frequency noise, etc.). The six realignment parameters were included in the general linear model GLM for all analyses as regressors of non-interest to account for biases in head movement correlations present during the experimental conditions.

#### Fixed effect analyses

Fixed effect SPM analyses were conducted for each subject. These analyses consisted of the following:

1. The contrast of post- relative to pre-training for the visual search trials compared to the rest trials (post-training relative to rest minus pre-training relative to rest) (See Fig 2B for timing of the various trial types). The event onset of the visual search trials for the GLM was taken as the time of the subject’s button response. The rest trials were included in the GLM from their onset to offset, lasting 10 seconds. Visual search trials without a button response were included as regressors of no interest in the GLM. **This contrast was designed to determine differences in brain activity after relative to before training**. The rest condition was used to ensure that differential activity post- relative to pre-training was not related to session effects, including unspecific tDCS effects. The same visual search task was used for pre- and post-training and therefore the conditions were matched for both visual input and motor output. The goal of this analysis was to determine brain activity showing task-related changes after training and active stimulation.
2. The contrast of target feedback for incorrect trials relative to target feedback for correct trials. This contrast was from data collected during the second session in which subjects were trained on the visual search task during real stimulation or sham stimulation (See Fig 2A for timing of the various trial types). During this training session an auditory feedback signal (‘ding’ sound-effect for correct responses and a ‘buzz’ for incorrect responses) was presented immediately with regard to the accuracy of their response. In addition, feedback of the true position of the target (target feedback—sphere appeared around target truck; See Fig 1C) was given at the end of the trials in which it was present. **Brain activity during target feedback presentation in particular was investigated as this will reveal signals associated with target feedback learning related processing that may be modulated by tDCS**. It is thought that the target feedback for the incorrect trials may provide more useful information important for attentional learning of the target features than correct target feedback trials. This is because there is an error signal present for incorrect trials but not for correct trials. The contrast under investigation was based on the onset of the target feedback modeled as an event for incorrect relative to correct trials. Other conditions included in the GLM consisted of the following: Ten-second rest trials; The full visual search epoch until the button press; The button press to the visual search and the simultaneous audio feedback; The button press to target-present feedback denoting incorrect hits (visual search response was a hit but the subject was attending to a distractor instead of the true target); Target feedback trials with no response or incorrect hits; All target absent trials. Contrasts were not conducted amongst these conditions so they can be thought of as regressors of non-interest extracting extraneous brain activity not related to target feedback processing. The contrast of target feedback incorrect versus correct trials was designed to specifically focus on activity related to training that is matched for visual input and motor output. Additionally, any unspecific effect due solely to tDCS should be cancelled-out as there is stimulation in both the incorrect and correct trials.

#### Random effect analyses

We conducted between subjects random-effects analyses (between subjects t-tests) using change in behavioral performance as a covariate of interest to determine performance related brain activity post- relative to pre-training that differs between the tDCS stim and sham groups. This was accomplished by using percent correct change in performance on the visual search task as a covariate of interest in the between subjects analysis of the contrast of post-training relative to rest minus pre-training relative to rest. The use of covariates allows us to investigate how the performance change (post-pre) of each subject is related to the change in overall activity on the visual search task (post-pre). Performance related brain activity specific to tDCS could be determined by comparing the stim group to the sham group. Essentially, we can determine why tDCS worked to increase performance for some subjects in the stim group and not for others. The use of covariates and the contrast of the stim vs sham group allow us to distinguish tDCS effects on performance rather than general learning effects resulting from training. Analyses were also conducted for the sham over the stim group.

Between subjects random-effects analyses were also conducted to determine differences in brain activity between the tDCS stim and sham groups during training. The contrast of stim over sham was conducted to determine differential brain activity between the two groups during the training session specifically related to target-error feedback for incorrect over correct trials that is hypothesized to be modulated as a result of tDCS stimulation. Analyses were also conducted for the sham over the stim group as well. Because it was later found that there was a significant difference in hit rate between the tDCS stim and sham groups in the training session we conducted an additional analysis using hit rate as a regressor of interest for the same target-error feedback contrast of stim relative to sham. The results of this contrast using a lenient threshold of p < 0.05 uncorrected was used as an exclusive mask for the primary analyses described above to ensure that the results were not due to differences in hit rate between stim and sham groups.

Correction for multiple comparisons (p < 0.05) across the entire brain was carried out using Monte-Carlo simulation of the brain volume to define a voxel contiguity threshold at an uncorrected significance level of p < 0.005 using the AFNI [61] 3dClustSim program that has been revised to address problems with cluster level correction for multiple comparison identified in [62]. Noise smoothness values using a spatial autocorrelation function were calculated using 3dFWHMx AFNI [61] program using the residual mean square image and the brain mask image from the random effects SPM analyses. Using 3dClustSim, 10,000 Monte-Carlo simulations were used to determine cluster size threshold correcting for multiple comparisons. Because of its role in error-based learning and because it is part of the dopaminergic system involved in facilitation of LTP [60], region of interest ROI analyses were conducted separately in the left and right basal ganglia. The WFU PickAtlas Tool Version 2.5.2 was used to make the ROI mask of the basal ganglia including the caudate, putamen, and pallidum.

Because we only included trials in which responses were given, it is possible that the number of trials between groups may differ and confound the brain imaging results. Analyses were conducted to ensure that there were no between group differences with regard to the number of trials included for each contrast of interest. For the target-error feedback contrast no significant differences (T = -1.28; p > 0.05 n.s.) in the number of trials were present between the tDCS stim (mean = 26.86 trials out of 30; SE = 0.69) and sham (mean = 27.93; SE = 0.53) groups. For the post- relative to pre-training contrast there were no significant differences for the following: 1. The post-training session (T = -1.66; p > 0.05 n.s.) between the tDCS stim (mean = 59.07 out of 60; SE = 0.48) and sham (mean = 59.86; SE = 0.10) groups. 2. The pre-training session (T = -0.41; p > 0.05 n.s.) between the tDCS stim (mean = 58.71; SE = 0.47) and sham (mean = 58.93; SE = 0.28) groups. And 3. The difference in the number of trials included for the post- minus the pre-training session (T = -0.88; p > 0.05 n.s.) between the tDCS stim (mean = 0.36; SE = 0.60) and sham (mean = 0.93; SE = 0.30) groups. Based on these results it is unlikely that the difference in the number of trials between the groups for the various contrasts confounded the brain imaging results.

### Transcranial direct current stimulation

TDCS was applied using a NeuroConn DC-Stimulator MR, which is a certified MRI compatible device, which allows for DC stimulation during magnetic resonance imaging. Conductive paste was applied to two (5.3 x 7.2 cm) rectangular-shaped MRI compatible rubber electrodes that were attached to the participant. The anode was placed over the right P4 (posterior parietal cortex (PPC)) according to the 10–20 International EEG System and held in place using a padded headband. The cathode was placed contralateral on the back half way between the shoulder and neck (trapezius muscle) that was held in place by the conductive paste and the weight of the participant as they lay in the supine position in the MRI scanner. An extracranial reference electrode placement was used to avoid any confounding effects on brain activity due to current density beneath the cathode [63].

Participants in the real stimulation group received 1mA current (maximum current dosage achievable with our MRI compatible tDCS unit) for a total of 30 minutes during the training session. Stimulation was started 5 minutes before the training began in order to ensure that the full modulatory effect of tDCS was active during task performance. The 30 minutes duration was sufficient time such that stimulation was always present during the task as well as during the acquisition of resting state activity used for a separate study [37]. Participants in the sham stimulation (control) group also received 1mA current but only for 30 seconds and then the unit was shut off. Given time constraints and the experimental setup in the MRI scanner it was not feasible to include a different stimulation area or polarity. However unspecific current/stimulation related factors were controlled for based on the experimental design (see above section on fMRI Data Collection and Analysis).

## Results

### Behavioral results

The between subjects t-test results for all dependent measures for the training session are given in Table 1. Only the hit rate was found to be statistically significant between the tDCS stim and sham groups.

**Table 1:** Behavioral results for the training session.

Performance Measure	Mean (SE) Stim	Mean (SE) Sham	T p<0.05
Percent Correct	64.86 (2.70)	67.94 (2.74)	-0.825 n.s.
Hit Rate	0.5178 (0.0382)	0.6262 (0.0337)	-2.21 *
False Alarm Rate	0.2227 (0.0284)	0.2672 (0.0403)	-0.94 n.s.
RT all Search	5.47 (0.260)	4.98 (0.230)	1.45 n.s.
RT HT&CR Search	5.40 (0.254)	4.94 (0.240)	1.36 n.s.
RT TP trials Search	5.03 (0.247)	4.48 (0.226)	1.705 n.s.
RT TP trials Target	1.29 (0.099)	1.13 (0.078)	1.27 n.s.

The behavioral results for various performance measures for the training session for the tDCS stim and sham groups. The between subjects t-tests were assessed using p < 0.05 with 26 degrees of freedom.

* = statistically significant at p < 0.05;

SE = standard error; RT = Response Time; HT = Hit; CR = Correct Rejection; TP = Target Present. The RT for the Target trials is the time spent observing the feedback before the button was pressed to continue to the next trial.

The t-test results for the pre-training session, the post-training session, and the post- minus the pre-training session are given in Table 2. No statistically significant differences were found between tDCS stim and sham groups for any of the behavioral measures (see Table 2). It is important to note that differences in response time between the tDCS stim and sham groups that could serve as a potential confound can be ruled out. This is because there were no statistically significant differences in any of the response time measures for the training session, the pre-training session, the post-training session, nor the post- minus pre-training session (see Tables 1 and 2). To determine whether there was an overall improvement in performance from pre- to post-training sessions for either group, additional paired t-tests were conducted for stim and sham groups. Percent correct performance post-training (mean = 72.06%; SE = 2.36) was significantly better relative to pre-training (mean = 64.26; SE = 2.64) for the tDCS stim group (t = 4.05; p < 0.05). There was also a significant improvement in percent correct performance post-training (mean = 73.02%; SE = 1.99) relative to pre-training (mean = 64.98%; SE = 2.47) for the sham group (t = 3.15; p < 0.05). This shows that performance was improved for both groups after training, however there was no statistically significant difference in the improvement in percent correct performance post- relative to pre-training between the groups as is reported in Table 2. All of the behavioral results reported above excluded trials in which there was no button response given. If we included these non responses as misses the results were essentially the same. There was a significant improvement in percent correct performance post-training (mean = 70.95%; SE = 2.02) relative to pre-training (mean = 62.86%; SE = 2.56) for the tDCS stim group (t = 3.85; p < 0.05). There was also a significant improvement in percent correct performance post-training (mean = 72.86%; SE = 2.02) relative to pre-training (mean = 63.81%; SE = 2.42) for the sham group (t = 3.53; p < 0.05).

**Table 2:** Behavioral results for the pre- and post- training sessions between tDCS stim and sham groups.

Performance Measure	Pre Mean (SE) Stim	Pre Mean (SE) Sham	Pre T p<0.05	Post Mean (SE) Stim	Post Mean (SE) Sham	Post T p<0.05	Post-Pre Mean (SE) Stim	Post-Pre Mean (SE) Sham	Post-Pre T p<0.05
Percent Correct	64.26 (2.64)	64.98 (2.47)	-0.21 n.s.	72.06 (2.36)	73.02 (1.99)	-0.33 n.s.	7.80 (2.00)	8.04 (2.65)	-0.08 n.s.
Hit Rate	0.5682 (0.0397)	0.6238 (0.0310)	-1.15 n.s.	0.5277 (0.0391)	0.5929 (0.0374)	-1.25 n.s.	-0.0465 (0.0399)	-0.0310 (0.0323)	-0.19 n.s.
False Alarm Rate	0.2813 (0.0270)	0.3234 (0.0521)	-0.74 n.s.	0.0900 (0.0168)	0.1337 (0.0294)	-1.34 n.s.	-0.1913 (0.0312)	-0.1896 (0.0464)	-0.03 n.s.
RT all Search	5.84 (0.314)	6.07 (0.221)	-0.61 n.s.	5.61 (0.272)	5.50 (0.179)	0.36 n.s.	-0.23 (0.212)	-0.57 (0.204)	1.20 n.s.
RT HT&CR Search	5.73 (0.324)	5.97 (0.259)	-0.59 n.s.	5.49 (0.266)	5.39 (0.191)	0.31 n.s.	-0.24 (0.232)	-0.57 (0.230)	1.07 n.s.

The behavioral results for various performance measures for the pre- and post- training sessions (and their difference post-pre) between the tDCS stim and sham groups. The between subjects t-tests were assessed using p < 0.05 with 26 degrees of freedom. SE = standard error; RT = Response Time; HT = Hit; CR = Correct Rejection.

### Brain imaging results

**Fig 3:**
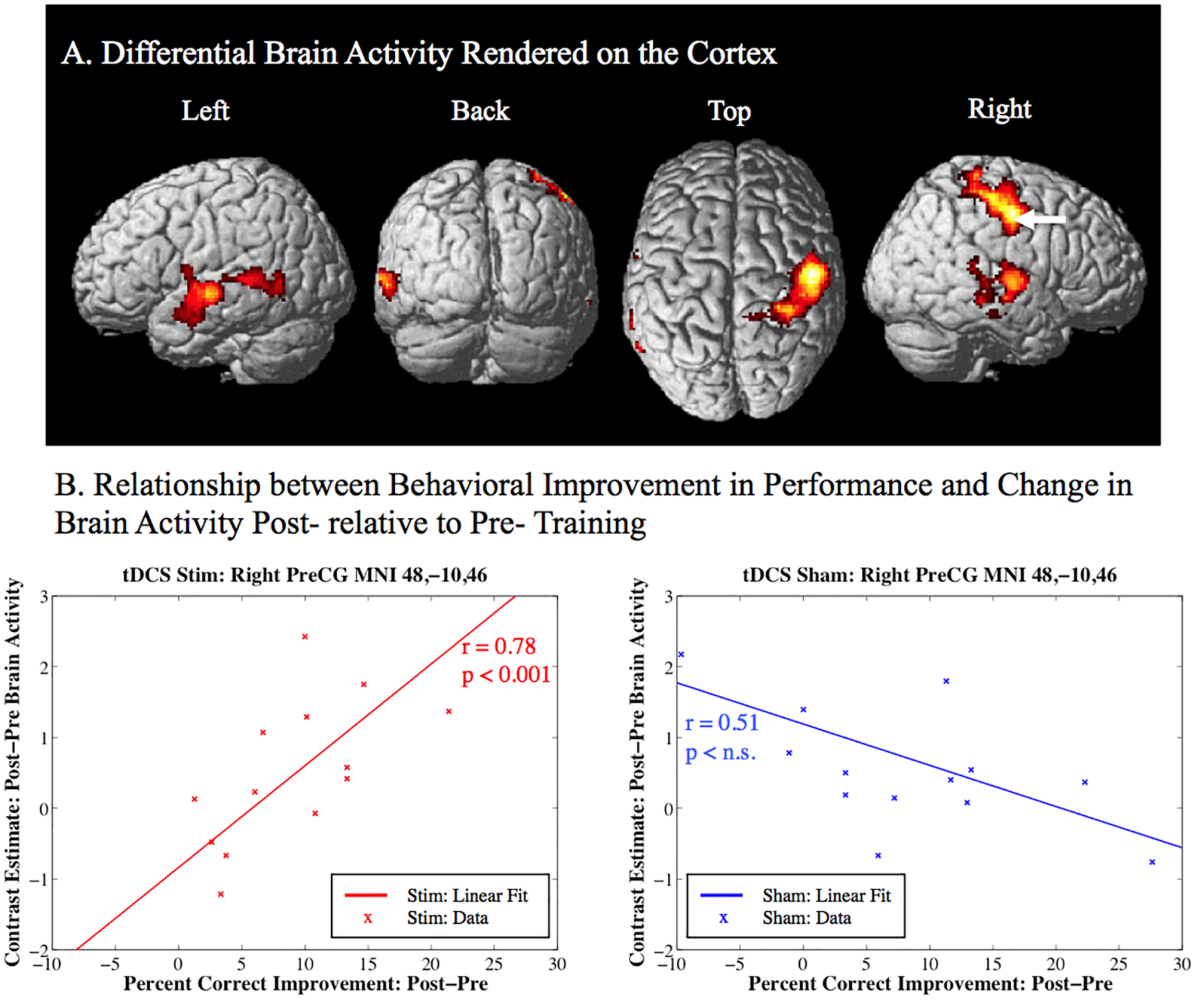
Behavioral improvement related activity post- relative to pre-training (Visual search—Baseline control) greater for the active stim group over the sham group. A. Clusters of differential activity included: right pre- and post-central gyrus, left superior and middle temporal gyrus, as well as right Heshyl’s gyrus and superior temporal gyrus. The results are corrected for multiple comparisons at the cluster level (p < 0.05) using Monte-Carlo simulation (corrected cluster extent threshold greater than 781 contiguous voxels over uncorrected significance threshold of p < 0.005). The white arrow indicates the location of the peak-voxel for the corresponding contrast. B. The linear relationship between behavioral improvement in performance (percent correct change) on the visual search task and the corresponding change in brain activity (contrast estimate: post-pre brain activity) post- relative to pre-training. PreCG = Precentral Gyrus.

**Fig 4:**
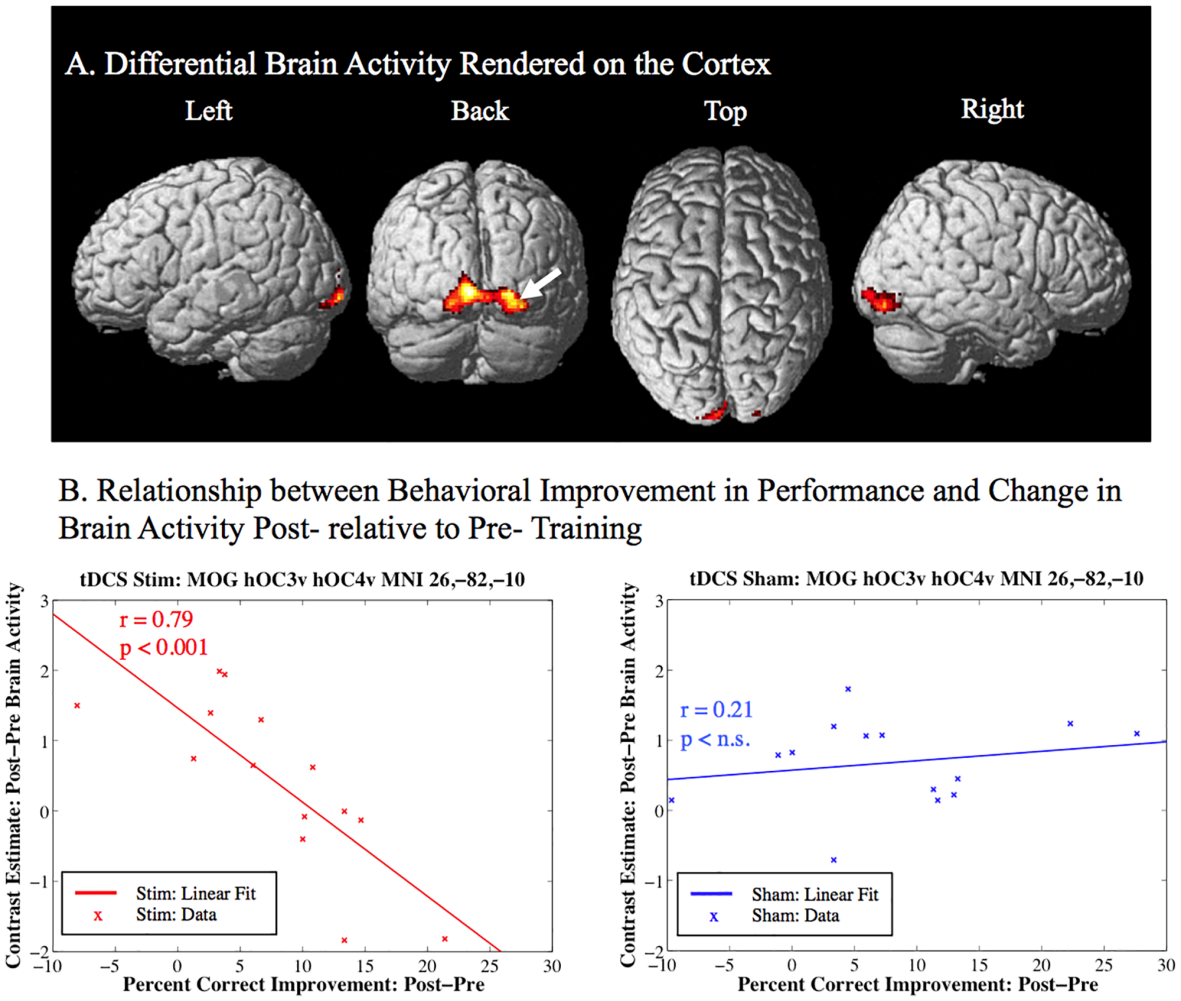
Behavioral improvement related activity post- relative to pre-training (Visual search—Baseline control) greater for the sham group over the active stim group. A. One cluster of significant differential activity encompassing the left and right calcarine sulcus, and the right the middle and inferior occipital gyrus including human occipital cortex visual processing areas V3 and V4. The results are corrected for multiple comparisons at the cluster level (p < 0.05) using Monte-Carlo simulation (corrected cluster extent threshold greater than 781 contiguous voxels over uncorrected significance threshold of p < 0.005). The white arrow indicates the location of the peak-voxel for the corresponding contrast. B. The linear relationship between behavioral improvement in performance (percent correct change) on the visual search task and the corresponding change in brain activity (contrast estimate: post-pre brain activity) post- relative to pre-training.

**Fig 5:**
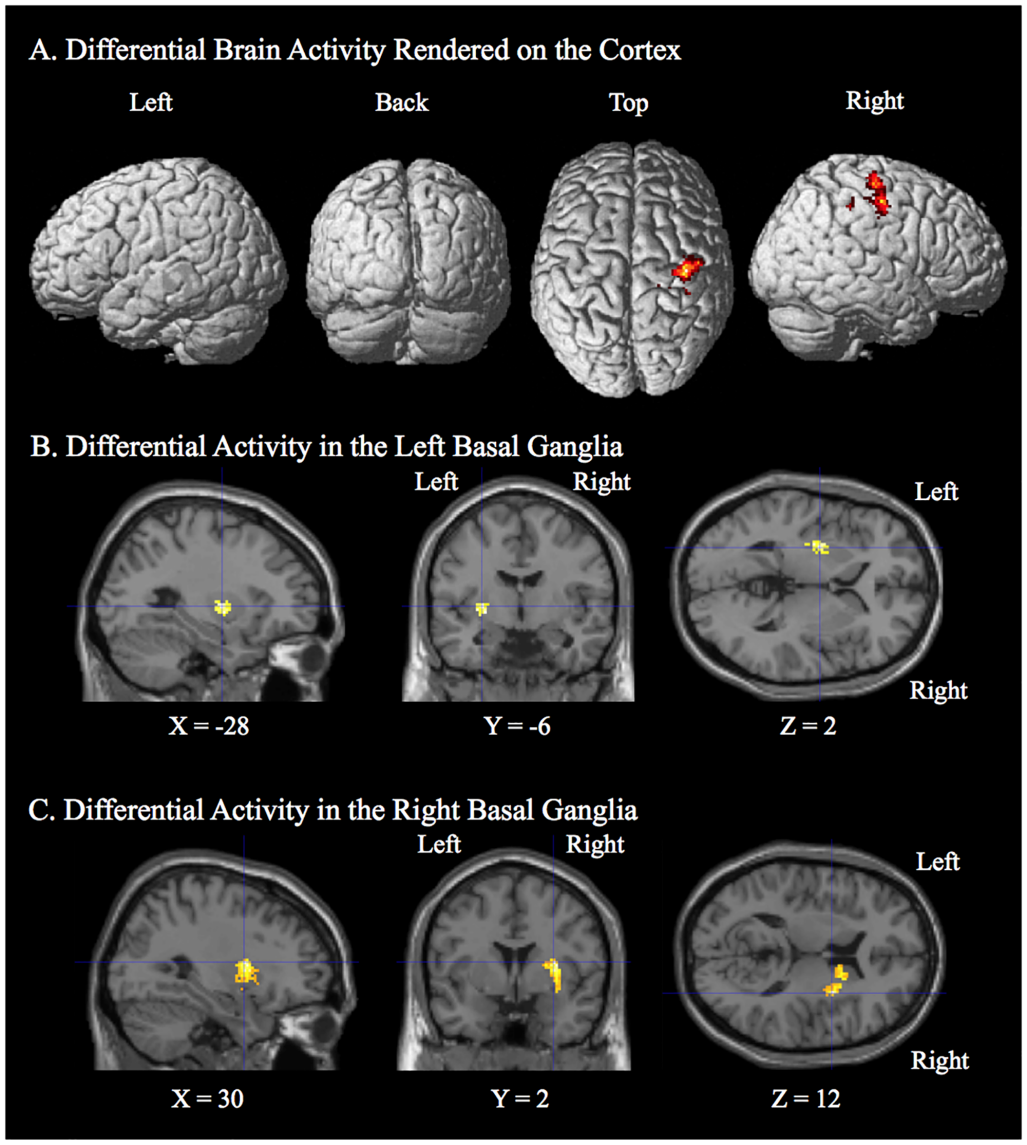
Brain activity unique to the active stim group over the sham group for target feedback of incorrect trials relative to correct trials during the training session. A) Differential activity rendered on the surface of the cortex. A single cluster of activity is present in the pre- and post-central gyrus and the premotor cortex. The results are corrected for multiple comparisons at the cluster level (p < 0.05) using Monte-Carlo simulation (corrected cluster extent threshold greater than 757 contiguous voxels over uncorrected significance threshold of p < 0.005). Differential activity determined by region of interest analysis in the left (B) and Right (C) basal ganglia rendered on MRI anatomical slices. Significant at p < 0.05 using Monte-Carlo simulation (corrected cluster extent threshold = 38 contiguous voxels for left basal ganglia and 35 contiguous voxles for the right basal ganglia, over uncorrected significance threshold of p < 0.005 within the region of interest). To ensure that activity wasn’t related to differences in hit rate between the stim and sham group but rather processing differences between incorrect and correct trials we employed an exclusive mask of activity present for the hit rate weighted F contrast of incorrect—correct for stim over sham at a lenient threshold of p < 0.05 uncorrected.

**Fig 6:**
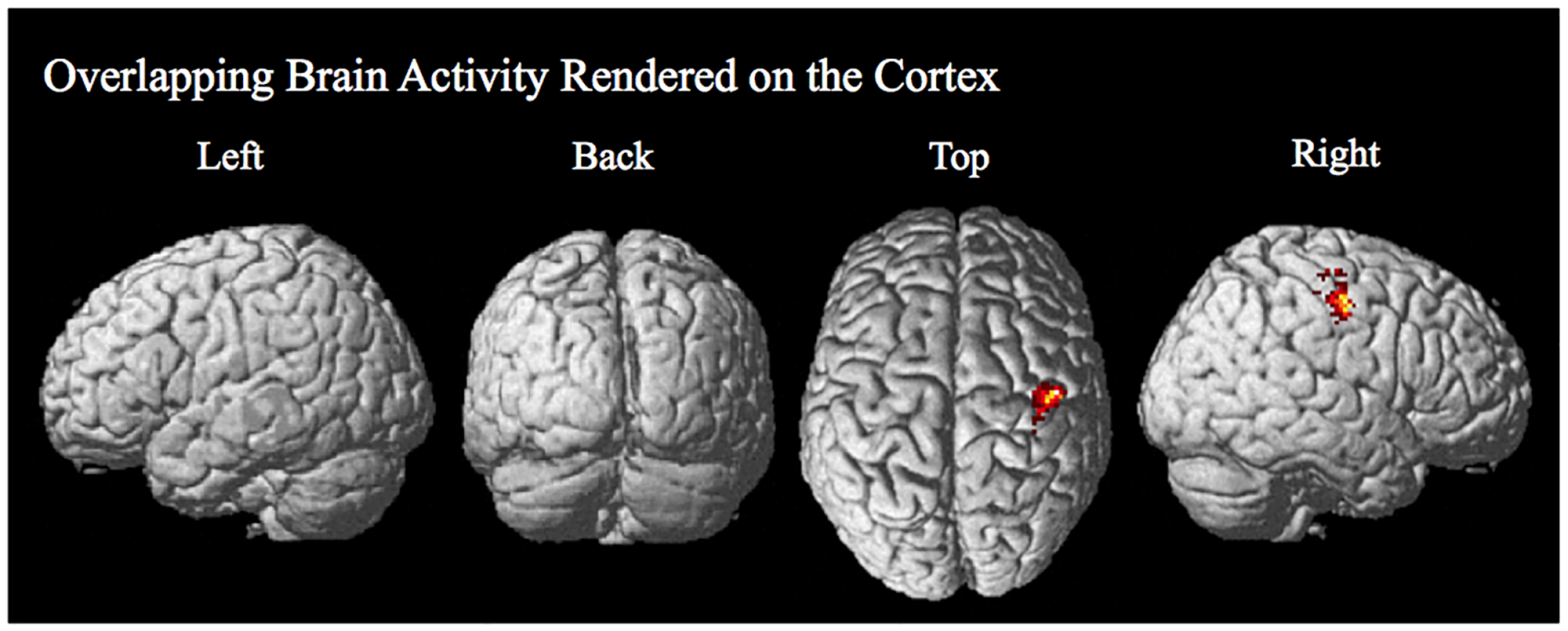
Overlap in behavioral improvement related activity with target feedback activity that is greater for the active stim group over the sham group. Overlap in significant voxels for the two analyses (Improvement Related Activity and Target Feedback Activity) are rendered on the surface of the cortex and are present in the pre- and post-central gyrus.

The brain imaging results for all the contrasts of interest are reported in Figs 3–6 and Tables 3–6. Correction for multiple comparisons (p < 0.05) was accomplished using Monte-Carlo simulation (Please see Methods section for more details). This correction method for multiple comparisons is the same for all brain imaging contrasts presented unless otherwise stated. Activated brain regions were identified using the SPM Anatomy Toolbox v1.8 [64] as well as Talairach Client after using the matlab min2tal function to transform from the MNI to the Talairach coordinate system.

**Table 3:** Behavioral improvement related activity post-training relative to preTraining (Stim—Sham).

Brain Region	MNI Coordinate x,y,z	T	Cluster Size
PreCG BA6,4	40,-12,48	3.92	1904
PreCG BA4,6	50,-12,44	4.82	1904
PstCG BA1,3	54,-14,44	4.97	1904
PstCG BA1	52,-18,56	4.92	1904
HG/STG BA22	52,-14,6	3.95	884
STG BA22	50,-8,0	3.71	884
M/STG BA21,22	-58,-6,-8	4.26	1077
M/STG BA21,22	-66,-46,6	4.21	1077

Brain regions showing significant behavioral improvement related activity for the stim over the sham group corrected for multiple comparisons at the cluster level (p < 0.05) using Monte-Carlo simulation (corrected cluster extent threshold = 781 contiguous voxels over uncorrected significance threshold of p < 0.005). The analysis involves using each subject’s change in behavioral performance as a covariate of interest for the post- relative to pre- training contrast. BA = Brodmann area; PreCG = Pre Central Gyrus; PstCG = Post Central Gyrus. HG = Heshyl’s Gyrus; STG = Superior Temporal Gyrus; Negative ‘x’ MNI coordinates denote left hemisphere and positive ‘x’ values denote right hemisphere activity.

**Table 4:** Behavioral improvement related activity post-training relative to pre-training (Sham—Stim).

Brain Region	MNI Coordinate x,y,z	T	Cluster Size
Calcarine Sulcus BA17	-4,-98,-4	5.25	999
MOG hOC3v, hOC4v	26,-82,-10	4.39	999
IOG BA17,18	22,-94,-6	3.89	999

Brain regions showing significant behavioral improvement related activity for the sham over the stim group corrected for multiple comparisons at the cluster level (p < 0.05) using Monte-Carlo simulation (corrected cluster extent threshold = 781 contiguous voxels over uncorrected significance threshold of p < 0.005). The analysis involves using each subject’s change in behavioral performance as a covariate of interest for the post- relative to pre- training contrast. BA = Brodmann area; MOG = Middle Occipital Gyrus; hOC3v = Human Occipital Cortex visual processing area V3; Human Occipital Cortex visual processing area V4; IOG = Inferior Occipital Gyrus. Negative ‘x’ MNI coordinates denote left hemisphere and positive ‘x’ values denote right hemisphere activity.

**Table 5:** Target feedback incorrect trials relative to target feedback correct trials (Stim—Sham).

Brain Region	MNI Coordinates x,y,z	T	Cluster Size
PMC BA6	34,-18,64	4.31	803
PreCG BA4,6	38,-12,46	4.29	803
PstCG BA3	24,-28,50	5.34	803
PstCG BA2	34,-32,42	4.69	803
Basal Ganglia Left	-28,-6,2	4.21	105*
Basal Ganglia Left	-30,-18,8	3.23	105*
Basal Ganglia Right	30,2,12	5.52	337*
Basal Ganglia Right	32,0,2	4.17	337*
Basal Ganglia Right	20,8,8	3.89	337*

Brain regions showing significant differential activity for incorrect relative to correct trials in the training session (session 2) for stim over sham corrected for multiple comparisons at the cluster level (p < 0.05) using Monte-Carlo simulation (corrected cluster extent threshold = 757 contiguous voxels over uncorrected significance threshold of p < 0.005). BA = Brodmann area; PMC = Premotor Cortex; PreCG = Pre Central Gyrus; PstCG = Post Central Gyrus; Negative ‘x’ MNI coordinates denote left hemisphere and positive ‘x’ values denote right hemisphere activity. To ensure that activity wasn’t related to differences in hit rate between the stim and sham group but rather processing differences between incorrect and correct trials we employed an exclusive mask of activity present for the hit rate weighted contrast of incorrect—correct for stim over sham at a lenient threshold of p < 0.05 uncorrected.

* Region of interest analyses using separate masks for the left and right basal ganglia from AAL anatomy database (caudate, putamen, pallidum left = 2124 voxels; right = 2207); Significant at p < 0.05 using Monte-Carlo simulation (corrected cluster extent threshold = 38 contiguous voxels for left basal ganglia and 35 contiguous voxles for the right basal ganglia, over uncorrected significance threshold of p < 0.005 within the region of interest).

**Table 6:** Overlap in behavioral improvement related activity to target feedback activity and connectivity during training that is unique to tDCS (Stim—Sham).

Brain Region	MNI Coordinates x,y,z	T	Cluster Size
PreCG BA4,6	48,-10,46	4.59	208
PstCG BA3	34,-26,46	3.85	208

Statistically significant behavioral improvement related activity post- relative to pre-training (Fig 3) that overlaps with target-error feedback activity (Fig 5). BA = Brodmann area; PreCG = Pre Central Gyrus; PstCG = Post Central Gyrus. Negative ‘x’ MNI coordinates denote left hemisphere and positive ‘x’ values denote right hemisphere activity.

#### Brain regions showing behaviorally related changes in activity post- relative to pre-training by tDCS

The random effects analysis investigated individual performance related activity for the contrast of visual search post- versus pre-training relative to the baseline rest condition, where each subject’s differential percent correct improvement for post- minus pre-training was used as a covariate of interest. Significant differential activity correcting for multiple comparisons for the stim group over the sham group is given in Table 3 and Fig 3. Clusters of significant activity (p < 0.05, corrected) include the right precentral gyrus, the right postcentral gyrus, the right superior temporal gyrus, and the left middle/superior temporal gyrus (See Table 3 and Fig 3). For the peak voxel, which resided in the right precentral gyrus (Fig 3A, arrow), the linear relationship between behavioral improvement in performance and the change in brain activity post- relative to pre-training is plotted in Fig 3B. Only the tDCS stim group shows a significant positive linear relationship (r = 0.78; p < 0.05; n = 14) between improvement in behavioral performance and the change in brain activity post- relative to pre-training.

For the sham over stim analysis there was a significant cluster of activity correcting for multiple comparisons within the left and right calcarine sulcus, lingual gyrus, and inferior occipital gyrus (See Table 4 and Fig 4). The linear relationship between behavioral improvement in performance and the change in brain activity post- relative to pre-training in the middle occipital gyrus (Fig 4A, arrow) is plotted in Fig 4B. For this region it can be seen that the tDCS stim group shows a significant negative linear relationship (r = -0.79; p < 0.05; n = 14) between improvement in behavioral performance and the change in brain activity post- relative to pre-training, whereas, the sham group does not show a significant linear relationship (r = 0.21; p > 0.05 n.s.; n = 14). For this brain region the sham-stim contrast is driven by reduced activity in the stim group associated with improved visual search performance.

#### Brain regions showing modulation in target feedback processing during training by tDCS

The results of the random effects analysis for the contrast of target feedback incorrect trials relative to target feedback correct trials for the tDCS stim group over the sham group are presented in Table 5 and Fig 5. A single cluster encompassing the premotor cortex the precentral gyrus, and the postcentral gyrus showed significant (p < 0.05, corrected) differential activity between the stim and sham groups (Table 5 and Fig 5A). A region of interest analysis was conducted separately in the left and right basal ganglia. The WFU PickAtlas Tool Version 2.5.2 was used to make a mask of the basal ganglia including the caudate, putamen, and pallidum. The region of interest analyses revealed significant clusters of activity (p < 0.05, corrected) in both the left and right basal ganglia (Table 5 and Fig 5B and 5C). It should be noted that there was no significant activity correcting for multiple comparisons for the sham over the stim analysis.

#### Overlap in regions differentially active for the tDCS stim over the sham group for brain activity related to individual differences in behavioral improvement and target feedback brain activity

To determine brain regions that are selectively modulated by tDCS that are related to training and individual differences in the improvement in performance on the visual search task, the overlap in brain regions found to be significant in the two analyses reported above was assessed. The results for the overlap in activity present for the stim over sham groups for these two analyses are given in Table 6 and Fig 6. The overlap is present in a cluster in the precentral and postcentral gyrus including the somatosensory cortex, premotor cortex, and primary motor cortex (Table 6 and Fig 6).

## Discussion

### Inter-individual variation in behavioral performance improvement correlates with differential post- versus pre- tDCS enhancement in task-specific activation

The results of this study elucidate potential neural modulatory mechanisms involved with task related individual differences in performance induced by tDCS. In accordance with the goals of this research, significant task related differences in brain activity between the stim and sham groups were identified. Specifically, a right hemisphere region on the border of the pre- and post-central gyrus including the somatosensory cortex, premotor cortex, and primary motor cortex were found to show the following: 1. Individual differences in behaviorally related enhancement in activity post- relative to pre-training for the tDCS stim over the sham group (See Fig 3 and Table 3). The degree of improvement in task performance was positively correlated with differential brain activity post- relative to pre-training only for the tDCS stim group. 2. Greater activity during target-error feedback processing for the tDCS stim over the sham group (simultaneous tDCS-fMRI). In the case of this experiment, the processes are specific to feedback following an incorrect response when a target was present (See Fig 5A and Table 5). 3. An overlap of differential activity related to individual behavioral improvement in performance and activity related to target error-feedback during training was identified in this brain region located on the border of the right pre- and post-central gyrus (See Fig 6 and Table 6). This overlap is consistent with tDCS inducing modulation of brain activity related to target-error feedback processing that is responsible for the enhanced brain activity correlated with improved performance observed in some individuals following training.

Individual differences in the extent to which tDCS affects learning is supported by the significant positive correlation between post-training brain activity in the precentral gyrus and behavioral performance (Fig 3B). In other words, participants in the tDCS stimulation condition with greater improvements in performance also showed higher increases in brain activity in this region. This relationship does not exist for the sham group anywhere in the brain.

### TDCS-induced decrease in task-specific activation in sensory cortex covaries with improvement in task performance across individuals

Another interesting finding of our study was the apparent decrease in improvement related activity in visual areas including human occipital cortex visual processing areas V3 and V4 as a result of tDCS stimulation (See Table 4 and Fig 4). These areas are thought to be involved in visual object perception, including early form processing in V3 [65–67]. Especially, V4 has been reported to play significant roles in object identification, such as figure-ground segregation from texture [68] and estimation of object surface properties (color: [69–72]; surface gloss: [73–74]). These processes are likely relevant for feature extraction in our visual search task that involves identifying a red truck amongst similar distractors in different orientations in a moving visual scene. While one may expect that better performance should be correlated with greater activity in these regions (consistent with the idea of tDCS induced excitability leading to LTP), this is the opposite of the relationship that we found. Alternatively, it may be the case that reduced activity in these visual processing regions with better performance for the tDCS stim group may reflect more efficient processing (less activity is required) consistent with [40–41, 45]. Less differential activity related to performance in these visual processing regions may result from tDCS induced modulation of processing in attentional networks including the frontal eye fields in the precentral gyrus (See Table 6 and Fig 6) that are related to optimizing orienting and saccadic eye movements to the demands of the visual search task.

### TDCS effect on premotor cortex may reflect modulation of attentional processes through LTP-like mechanisms during visual search training

In support of our hypothesis that tDCS modulates attentional processes during visual search training, previous research has shown that the same region in the pre- and post-central gyrus that we found to show overlap in all of our analyses (see Fig 6 and Table 6) is involved with visual attention and orienting [75–77]. Studies have shown that there is a large overlap of visual attention and oculomotor neural networks in regions found in the temporal, parietal, and frontal lobes [75, 78]. A major node of activation in the frontal lobe found in these studies for both covert shifts in attention and planned saccadic eye movements is the premotor cortex; specifically, the precentral gyrus/sulcus. Within this region, areas of activation are found that respond selectively to either or both covert attention shifts and planned eye movements. Following these results, Corbetta and colleagues identified the precentral gyrus/sulcus as the human homologue for the frontal eye fields (FEFs) and the most anterior node of the top-down attention network [56–57]. While the location of the human FEFs has been difficult to pinpoint, a recent meta-analysis published by [79] also supported this assertion by identifying the human FEFs as being located in Brodmann area 6 somewhere between anterior precentral gyrus and precentral sulcus near the superior frontal sulcus. The premotor regions identified in these previous studies overlap with the cluster of activation revealed in our results to be associated with individual differences in improved learning and performance in the tDCS group during visual search. This provides evidence that tDCS is modulating attentional processes during visual search training for some individuals. It has been conjectured that the active facilitative effects that anodal tDCS has on various cognitive components, such as attention, may be a result of overall lowering of local neural firing threshold of areas near the site of stimulation [2] as well as areas functionally associated with the site of stimulation [2, 38, 80]. As a result, there is an increase in activity dependent synaptic potentiation which elicits mechanisms of long-term potentiation (LTP) in the affected neural circuits resulting in a strengthening of connections between synapses relevant to task performance [2, 9, 10]. Evidence of long-term effects of tDCS on LTP/LTD is supported by magnetic resonance spectroscopy (MRS) experiments demonstrating that anodal tDCS stimulation increases glutamatergic concentration [81] and conversely that cathodal tDCS stimulation reduces glutamatergic concentration [50]. TDCS induced LTP may explain learning performance after-effects of tDCS as was observed through increased brain activity for some individuals in this study, which results in a continued increased excitability after the cessation of anodal stimulation for at least an hour [12].

### TDCS modulation on neural processes for error-feedback learning may underlie long-term facilitative effect of tDCS

It is also interesting to point out that this region in the pre- and post-central gyrus found to overlap in all our analyses also shows greater resting state activity as measured by fractional amplitude of low frequency fluctuations for the stim over the sham group during concurrent tDCS stimulation [37]. Additionally, resting state functional connectivity during tDCS stimulation from the site of stimulation (precuneus) to the substantia nigra, was found to be positively correlated with individual differences in visual search performance [37]. This is interesting in that the substantia nigra and basal ganglia (found to be differentially active for stim > sham for target-error feedback training: see Fig 5 and Table 5) is known to be part of the dopaminergic system and that dopamine is known to facilitate LTP [60].

### Potential limitations and confounds related to concurrent tDCS and fMRI paradigm

As with all brain imaging studies, there are many potential limitations and confounds that need to be addressed. One potential limitation of this study is that the changes we see in BOLD response may not be a result of changes in task-relevant neural activity, but rather may be a result of changes in cerebral perfusion or noise induced by tDCS (this is only a potential problem for session two in which concurrent tDCS and fMRI was applied). Previous studies using concurrent tDCS and fMRI have suggested that tDCS induced distortions on fMRI signal-to-noise ratio SNR are minimal [34, 82]. In addition, because there were within subject control conditions for all contrasts these types of potential confounds are controlled for. In terms of the performance-related post- relative to pre-training activity analysis we employed there is no reason why changes in cerebral perfusion or noise induced by tDCS would correlate with behavioral performance. It is much more likely that the performance-related brain activity we observed is a result of changes in task-dependent neural activity resulting from tDCS stimulation.

An additional confound that we did not test was whether the radio frequency and gradients associated with fMRI EPI scanning influences the tDCS current. The NeuroConn DC-Stimulator MR that we used for this experiment includes an RF filter module with MRI compatible cables and electrodes. These components help to prevent effects of RF on tDCS current. In addition, we avoided cable loop formation in the setup that may result in gradient induced currents. Furthermore, the fMRI compatible cables used for tDCS had a high resistance (5kΩ), which should also decrease the induced current avoiding potential effects of MRI on tDCS. Although we do not believe it to be the case, since we did not measure the current during stimulation, we cannot rule out the possibility that the tDCS current could have been reduced or modulated such that its enhancement effects on behavior were diminished.

## Conclusions

The main purpose of this study was to identify tDCS induced brain activity associated with individual differences in enhancement of behavioral performance that may shed light on the growing number of studies disputing the reliability of tDCS. This was accomplished by using an fMRI-compatible tDCS device that allowed us to concurrently apply DC stimulation while measuring brain activity during visual search training and performance. A cluster was identified during target-error feedback in a brain region believed to contain the FEFs that is a major node in the top-down attention network. This suggests an active effect of tDCS on task-relevant networks during training. In addition, we identified a significant relationship between individual differences in visual search improvement and brain activity following the training session (after-effects) in the same attentional brain region for the real stimulation group that did not exist for the sham stimulation group despite not observing differences in behavioral performance at the group level. Specifically, individuals who had greater increases in visual search performance also displayed increased brain activity in the FEFs. This individual difference in the increase in activity during and after stimulation within task-relevant networks in areas not directly beneath the electrode is consistent with long-range tDCS induced LTP-like mechanism resulting in synaptic strengthening within these networks. Furthermore, the decrease in activity in visual areas with greater individual differences in improvement of visual search performance may represent more efficient processing resulting from processing in the FEF. Our findings together suggest that the apparent lack of reliability found in tDCS research in healthy adults may result from individual differences in the efficacy to which tDCS can induce long-term modulation of processing seen in after-effects in relation to enhanced task performance. The behavioral and fMRI data used for the results of this study are available as supplementary data S1 File.

## Supporting information

S1 File Behavioral and fMRI data. (ZIP)
